# Oral Health-related quality of life after coronectomy for impacted mandibular third molar in the first postoperative week

**DOI:** 10.4317/medoral.24506

**Published:** 2021-05-23

**Authors:** Jacco G Tuk, Lily E Yohannes, Jean-Pierre TF Ho, Jerome A Lindeboom

**Affiliations:** 1 Department of Oral and Maxillofacial Surgery, Amstelland Hospital, Amstelveen, The Netherlands; 2 Department of Oral and Maxillofacial Surgery, Amsterdam UMC, University of Amsterdam, Amsterdam The Netherlands

## Abstract

**Background:**

Coronectomy of a mandibular impacted third molar is a surgical treatment to minimize the risk for inferior alveolar nerve damage. We aimed to determine whether this procedure affected the oral health-related quality of life (OHRQoL) within the first postoperative week.

**Material and Methods:**

This prospective study included 50 patients that underwent a coronectomy for an impacted mandibular third molar. The patients completed the Oral Health Impact Profile-14 (OHIP-14) questionnaire and questions about pain and analgesic intake on every day during the first postoperative week.

**Results:**

Mean OHIP-14 scores were highest during the first three postoperative days; the highest mean score (26.40, SD: 8.67) was observed on the first postoperative day. Mean OHIP scores gradually declined during the first postoperative week, and the mean OHIP-14 score was 9.82 (SD: 9.15) on the seventh day. Physical pain was the highest contributor to the overall OHIP-14 score. Pain gradually declined with time; the lowest mean pain score (3.38, SD: 2.2) was observed on the seventh day. OHIP-14 and pain scores were not significantly different between sexes or between different grades of impaction. OHIP-14 scores were positively correlated with pain scores.

**Conclusions:**

A mandibular third molar coronectomy had a strong effect on patient OHRQoL, particularly during the first three postoperative days.

** Key words:**Third molars, coronectomy, pain, OHRQoL.

## Introduction

Surgical removal of the mandibular third molar is a very common oral surgical procedure. Postoperative inflammatory conditions, like alveolar osteitis and surgical site infections, are frequent complications after this procedure, but they are typically easy to manage. A less common, but more serious complication is an inferior alveolar nerve (IAN) injury, which can lead to a neurosensory deficit. In 1-3.6% of IAN injuries, the neurosensory disturbance is permanent (1-3). This can cause long-term effects, such as persistent sensory loss, chronic pain, and depression (4). The risk of damaging the IAN is high during surgical removal of a third mandibular molar, due to the close relationship between the molar roots and the IAN.

The IAN is located deep in the mandible; thus, a coronectomy can minimize the risk of IAN injury (5-11). The fundamental objective of a coronectomy is to prevent trauma to the IAN by removing only the crown of an impacted mandibular third molar. Thus, the roots remain in place, and the IAN is untouched (12).

Previous reports on the mandibular third molar coronectomy were mainly focused on the surgical technique, root migration, postoperative IAN function, socket healing, and postoperative inflammatory parameters (3,6,13). Little emphasis has been placed on the postoperative quality of life (QoL). As in any surgery, the coronectomy of a mandibular third molar causes tissue damage, and as such, it will have an impact on the oral health-related quality of life OHRQoL.

The present study aimed to investigate whether an impacted mandibular third molar coronectomy would affect the OHRQoL during the first postoperative week. We surveyed patients with the Oral Health Impact Profile-14 (OHIP-14) questionnaire. Previous studies have demonstrated the effect of surgical removal of mandibular third molars on OHRQoL with the OHIP-14 questionnaire, but no study focused on the mandibular third molar coronectomy (14-15). In addition, we assessed postoperative pain, swelling, trismus, alveolar osteitis, and infection in the week after a third mandibular molar coronectomy.

## Material and Methods

- Participants

Eligible patients were referred by their dentist to the Department of Oral and Maxillofacial Surgery of the Amstelland hospital in Amstelveen, The Netherlands, for removal of an impacted mandibular third molar. Patients with asymptomatic impacted mandibular third molars that underwent a coronectomy between January 2019 and December 2019 were included. Inclusion criteria were age 18 years or older, healthy (American Society of Anesthesiologists (ASA) 1), willing to participate, and able to read, understand, and answer the questionnaire. Exclusion criteria were: known allergies to ibuprofen or chlorhexidine; smoker; periodontitis; a medical history involving renal failure, blood diseases, or chronic liver disease; taking anti-aggregants or corticosteroids, currently, or in the 15 days prior to surgery; breastfeeding or pregnant; local infection, preoperatively or in the 15 days prior to surgery; previous radiation therapy to the maxillofacial region; uncontrolled diabetes; taking antibiotic prophylaxis for endocarditis; or any local pathology.

This prospective study was reviewed and approved by the institutional Medical Ethics Committee of the Amsterdam University Medical Center. The study was conducted in accordance with Good Clinical Practice and the Declaration of Helsinki, as amended in Somerset West, Republic of South Africa, in 1996. Patients were provided with information to explain the study, and all patients consented to participate in the study. Patients also agreed to attend two appointments (the surgery and a control visit). All patients were fully informed about the surgical procedure, postoperative care, possible complications, and follow-up examinations. Each patient was informed that they had the opportunity to withdraw from the study at any time, without consequences regarding the treatment.

- Study procedure

This study included 50 patients. Preoperatively, patient demographic and medical information was recorded, and the patients were labeled patient 1 to patient 50, to ensure confidentiality of patient information during the study. We recorded the location of the impacted third molar, and we performed an X-ray orthopantomogram to assess the degree of impaction (Pell and Gregory’s classification). We also recorded the proximity of the IAN to the third molar roots. With 3-dimensional computed tomography, we confirmed the relationship between the IAN and the roots of the impacted mandibular third molar.

- Surgical procedure

The impacted mandibular third molar coronectomy was performed with the patient under local anesthesia. All surgeries were performed by one surgeon in a standardized fashion, with a similar technique in all cases. All patients received a standardized, mandibular nerve block injection, with additional local infiltration of the buccal nerve. The location, temperature, type, and amount of anesthetic (40 mg articaine/hydrochloride with .01 mg epinephrine, administered with a 1.7-mL syringe, Ultracain D-S forte; Sanofi-Aventis, Netherlands BV, Gouda, the Netherlands), and the type of needle (27 gauge/.40 × 35 mm) were all standardized, according to the hospital protocol. A triangular flap was used in all patients. Briefly, an incision was started at the distobuccal edge of the second molar, then dropped at a slight oblique angle, and then curved forward into the mandibular vestibule. The second part of the incision started from the mandibular ramus and ended at the distobuccal aspect of the second molar. Any bone overlying the crown of the impacted third molar was removed with a round surgical bur, which exposed the cementoenamel junction of the tooth. Next, a fissure bur was used to separate the crown from the roots. The root was shortened to 3-4 mm below the bony margin and checked for mobility. Copious irrigation with sterile saline was performed with rotary instrumentation. Dental follicular soft tissue was removed, and the socket was thoroughly irrigated with saline. The surgical site was primarily closed with 3/0 Undyed Vicryl Rapide (Ethicon, Somerville, MA, USA). Immediately after surgery, the details of the procedure were recorded.

- Postoperative management

After surgery, all patients were instructed to bite on a gauze for 30 min. They were also instructed not to rinse or spit during the first 24 h postoperatively. Ibuprofen (600 mg Brufen, Abbot BV, Hoofddorp, the Netherlands) was prescribed three times a day. No postoperative antibiotics were prescribed. The day after surgery, patients began rinsing the mouth with a 0.12% aqueous chlorhexidine mouth rinse for 1 min twice per day for 7 days. Patients were given verbal and written postoperative instructions, and they were recalled for follow-up at 1 week.

- Follow-up

One week after surgery, patients were examined to assess surgical site wound healing and to check for alveolitis and wound infection. At that time, the completed OHIP-14 questionnaires were collected.

- Outcome measurements

The outcome measurements included the OHIP-14 score, the pain score, based on the numeric rating scale (NRS) and the daily analgesic intake. OHRQoL was assessed with the OHIP-14 questionnaire. It involved the following parameters: problems pronouncing words, altered sense of taste, difficulty in chewing, pain/aching, worry about dental problems, psychological discomfort, problems affecting the diet, interruptions in meals, difficulty relaxing, feeling embarrassed, feeling irriTable, job-related difficulties, less satisfaction in life, and functional inabilities. The short form of the OHIP-14 consisted of 14 items within 7 domains, including: functional limitations, physical pain, psychological discomfort, physical disability, psychological disability, social disability, and handicaps (15-16). Patients rated each item on a 5-point scale, with 4=very often, 3=fairly often, 2= sometimes, 1=hardly ever, and 0=never. The total score ranged from 0 (minimum impact) to 56 (maximum impact). Scoring high on the OHIP-14 questionnaire indicated that the surgery had a strong impact on the OHRQoL. Pain assessment was measured by rating pain intensity with an 11-point NRS, which ranged from 0 (no pain) to 10 (worst possible pain). Patients self-rated their pain on each day of the first postoperative week (14-16). The questionnaires were completed daily at the end of the day. At the 1-week control visit, an independent assessor evaluated wound infection, alveolitis, and the sensory function of the IAN. The patients were assessed for sensory disorders, such as pain, numbness, dysesthesia, or paresthesia, based on a 2-point discrimination and static light touch detection test (3).

- Data management

All patient data on infection, alveolitis, analgesic intake, pain scores and OHIP-14 scores were collected between January 2019 and December 2019 and imported into a database. The data also included two demographic variables: the age at surgery (years) and sex. Gregory and Pell’s classification of the third molar position was used to describe the degree and type of mandibular impaction.

- Statistical analysis

Conventional descriptive statistics were performed to characterize the patient sample. The Shapiro-Wilk test showed that all the outcome variables in this study were normally distributed (*p*>0.05). Repeated measures ANOVA was used to assess the mean differences of OHIP- and pain scores over time from day 1 to day 7. If the overall *p-value* of the repeated measures ANOVA was smaller than 0.05, pairwise comparison was used to test differences between any two time points. Pearson’s correlation test was performed to analyze correlations between different variables. The independent sample t-test was used to determine if there was a significant difference in OHIP-14 scores, pain scores and analgesic intake between the male and female variables on each postoperative day.

## Results

Data from 50 patients were available for analysis, including 13 (26%) males and 37 (74%) females. The mean ages were 25 years (range: 19 to 35) for males and 25 years (range: 18 to 36) for females. The treated third molar was on the left side in 30 patients and on the right side in 20 patients. The treated molar alignments included 60% mesioangular, 24% horizontal, and 16% vertical (Table 1). The degree of impaction varied from grades 1A to grade 3B, according to Gregory and Pell’s classification.

**Table 1:**
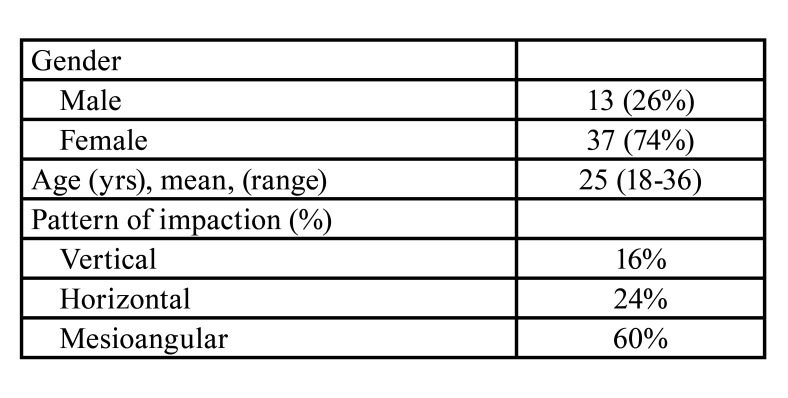
Patient demographics and mandibular third molar characteristics.

- OHIP-14 and Pain scores

Table 2 and Table 3 show the mean pain- and OHIP-14 scores on each postoperative day. On the first postoperative day, pain was the highest (mean score; 6.40 SD 2.07). Pain gradually declined with time, being the lowest on the seventh day (mean score: 3.38, SD 2.24).

**Table 2:**
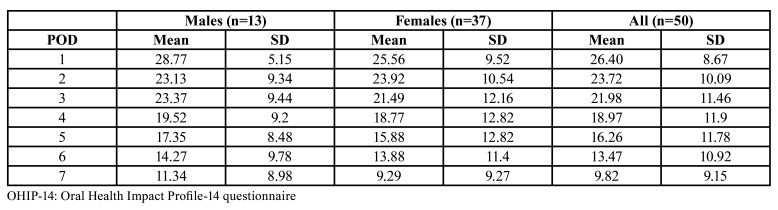
Means and standard deviations (SD) of total OHIP-14 scores for males, females, and total samples on postoperative days (POD’s) 1 to 7 (n=50).

**Table 3:**
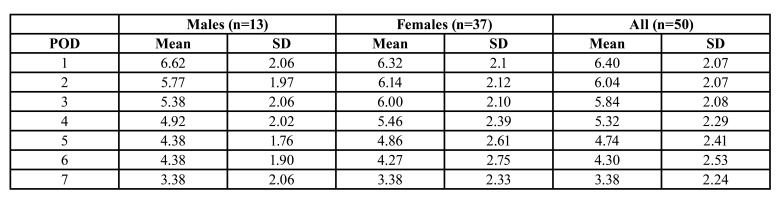
Means and standard deviations (SD) of total pain scores for males, females, and total samples on postoperative days (POD’s) 1 to 7 (n=50).

Results from the repeated measures ANOVA test showed this decline in pain score was significant (*p*<0.01).

The repeated measures ANOVA test also showed this decline over time in the mean OHIP-14 scores (*p*<0.01), which was statistically significant on each postoperative day (*p*<0.05). No significant differences between males and females in the total OHIP-14 scores and the pain scores on each postoperative day were found.

- Correlation between pain and OHIP-14 scores

**Figure 1:**
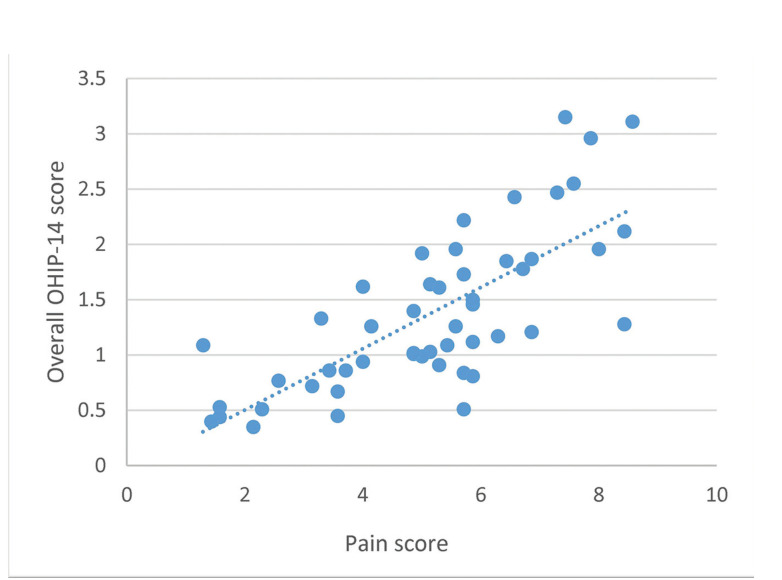
Scatterplot of pain scores vs. OHIP-14 scores. The dotted line shows a significant positive correlation (r=0.743, n=50, *p*<0.05).

The correlation between the pain scores and the overall OHIP-14 scores were analyzed with Pearson’s correlation. We found a positive correlation between these variables (r=0.743, n=50, *p*<0.05; Fig. 1).

- Pell and Gregory classification

Analyzing the means of OHIP-14 scores and pain scores between the different categories of the third molar impaction grades for any postoperative day, we found that the impaction grade did not influence the OHIP-14 and pain scores.

- Analgesic intake

No differences were found for the postoperative mean analgesic intake, except for the first day were more painkillers were used by females than males. However, this difference was not statistically significant.

- Postoperative complications

One case of postoperative infection occurred during the first postoperative week. After 3 days, an abscess appeared and was drained. Subsequently, the patient was given amoxicillin 3 times per day for 5 days. Postoperative alveolitis did not occur in any patient, and no sensory disturbances of the IAN were detected at the 1-week follow-up visit.

- Coronectomy versus surgical removal OHIP-14 and pain scores

Table 4 compares the OHIP-14 and pain scores from the present study with data from an earlier prospective cross-over, randomized controlled study where patients underwent surgical removal of an impacted third mandibular molar (14). The data from the control group were compared to the coronectomy group in the present study. The basic characteristics of the two groups was comparable (mean age of 25 years, all impacted third molars required bone removal).

**Table 4:**
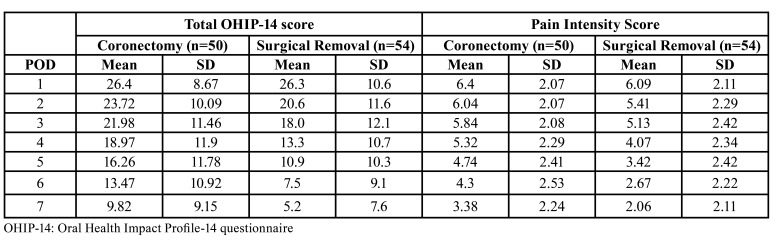
Comparison of means and standard deviation (SD) of total OHIP-14 scores for coronectomy versus surgical removal of impacted mandibular third molars on postoperative days (POD’s) 1 to 7.

The mean OHIP-14 scores were comparable for the 1st postoperative day but were higher in the coronectomy group for the remainder of the week. Mean pain scores were higher in the coronectomy group compared to the surgical removal group for each day of the postoperative week.

## Discussion

This study investigated the OHRQoL in patients after a coronectomy for an impacted mandibular third molar. Mean OHIP-14 scores were highest during the first three postoperative days and gradually declined during the first postoperative week. Pain was the highest on the first postoperative day and declined gradually. Pain occurs with tissue injury, which leads to the formation of prostaglandins from the enzymatic degradation of arachidonic acid in the lipid membrane by cyclooxygenase (COX) (17). Then, as the membrane lipids are restored by tissue repair mechanisms, pain is gradually reduced each day. Analgesics inhibit the COX enzyme, and thus, inhibit the production of prostaglandins, which minimizes pain sensation. Therefore, analgesics affect the pain score. In the present study, patients received 600 mg ibuprofen 3 times per day, and when necessary they were instructed to combine the 600 mg of ibuprofen with 1000 mg of acetaminophen (paracetamol). The analgesic intake in this study was highest on the third day (mean: 3.76) and lowest on the seventh day postoperative (mean: 1.86).

In the present study, we found no significant differences in OHIP-14 and pain scores between males and females. Our findings were in contrast with those of Fillingim *et al*. (18), who reported that some forms of pain were more prevalent among females than among males. They found that women experienced more pain than men in oral‑related issues, such as tooth pain and jaw joint pain (18). Another study found that women reported more pain then men after an invasive oral surgical procedure (19). However, other studies reported no differences in pain between the sexes after oral surgery (20-21).

We found that the degree of impaction, according to Gregory and Pell’s classification of the third mandibular molar, did not impact the pain score. In contrast to the expectation that surgery on more deeply impacted mandibular third molars would have a more significant impact on the OHRQoL, we did not find any significant difference in the degree of pain experienced for different degrees of impaction. Nevertheless, we found a high positive correlation between physical pain and the OHRQoL, consistent with our findings regarding the pain domain of the OHIP-14 questionnaire (22). Indeed, ‘physical pain’ was the highest contributor to the overall OHIP-14 score. Therefore, the pain score could be used to predict the effects of pain on the QoL after a coronectomy.

After the coronectomy, patients exhibited a reduced ability to chew and enjoy food. They experienced limited mouth opening and had to adjust their diet. In particular, during the first few postoperative days (days 1 to 3), patients had difficulties in opening the mouth or chewing. Most patients required liquefied or soft foods that could be swallowed without much chewing.

An important question is whether a coronectomy of an impacted mandibular third molar might impact the QoL or pain score more than the surgical removal of an impacted mandibular third molar. Only one previous study reported on QoL after a coronectomy. Manor *et al*. (23) compared 34 patients that underwent a coronectomy and 35 that underwent surgical removal of the mandibular third molar. Similar to the present study, they administered a OHRQoL questionnaire to patients during the first postoperative week. They found no differences in QoL scores between the groups. For both groups, the first three days were the most difficult, regarding pain, swelling, and oral and general functions. Comparing the OHIP-14 and pain scores of the present study with an earlier study on surgical removal of impacted mandibular third molars, we found higher scores for the total OHIP-14 and pain after patients underwent a coronectomy (14). A potential explanation of these differences in the OHIP-14 and pain scores between these studies might be that, in some cases, a coronectomy might require greater surgical invasiveness compared to a complete surgical removal. Indeed, Zola (24) pointed out the concern that the postoperative course was more protracted for a coronectomy than for a surgical removal. One reason for this difference might have been that a larger flap and greater bone removal was required to complete the coronectomy compared to the surgical removal. Consequently, patients might have experienced greater immediate postoperative discomfort after a coronectomy. In addition, after the coronectomy, the exposure of pulp tissue might increase the risk of infection or prolong sensitivity or pain. Previous studies have described increased pain in patients after a coronectomy compared to a surgical removal (7-8). However, other studies found that the incidences of pain and swelling after a coronectomy were lower than those reported after the surgical removal of a partially or completely impacted mandibular third molar (6,12).

In the present study, no patient experienced sensory impairment of the IAN after the mandibular third molar coronectomy. Previously, a randomized study compared surgical removals to coronectomies in 128 patients. They found that 19% of the surgical removal group sustained IAN damage and no IAN symptoms were reported among the successful coronectomies (25). Other studies confirmed that no IAN injury occurred with a coronectomy (3,8,10). In the largest prospective study on coronectomies, among 612 coronectomies of impacted mandibular third molars, the prevalence of IAN deficits was only 0.16% (11).

In the present study, only one patient experienced a postoperative infection: an abscess occurred on the third postoperative day. The abscess was drained, and amoxicillin was given 3 times per day for 5 days. Postoperative infection rates after a mandibular third molar coronectomy have varied between 3.2 and 5.8 % (6,25). The infections were always treated with antibiotics and debridement. Leung and Cheung [2016] showed that, among 612 coronectomies, infections occurred in 2.9% (11). However, Cilasun *et al*. (8) found no postoperative infections in a coronectomy group.

In a coronectomy, the roots remain in place; over time, this situation can lead to symptoms and pain. Due to this potential complication, some patients and oral surgeons might hesitate in selecting this treatment (5). On the other hand, the significant reduction in the risk of neurosensory disturbances after a coronectomy can offset the risk of a future second surgery; indeed, the need to remove migrated roots was only reported in 3.3% of cases (11). The coronectomy is typically performed on healthy teeth without pathology; consequently, the retained roots should pose less of an issue compared to teeth with some form of pathology, which is frequently observed in erupted teeth.

In the present study, no cases of alveolitis were observed. The incidence of dry socket after a coronectomy was previously reported to be relatively low, due to the facts that the wounds were small, little alveolar bone was exposed, and primary wound closure was performed (7). Leung & Cheung (6) reported no cases of dry socket in a coronectomy group, compared to 2.8% cases of alveolitis in a surgical removal group. In a later study, among 612 coronectomies on lower third molars in 458 patients, only one coronectomy (0.16%) resulted in a dry socket in the first postoperative week (11). However, Renton *et al*. (25) reported a 12.1% incidence of postoperative alveolitis in a coronectomy group, which was comparable to the 9.6% postoperative alveolitis observed in a surgical removal group. In that study, the high incidence of alveolitis observed after a coronectomy might have been due to the fact that the mucoperiosteal flaps were replaced with a single suture; thus, compared to other studies, they did not achieve a ’water-tight’ closure. Another explanation might be that, in that study, a high proportion of patients were treated for difficult, deeply impacted teeth with pericoronitis (25).

The main limitation of this study was that we included only 50 participants. Although the procedure was similar in all cases, a small sample size increases the margin of error and affects the reliability of the study results.

In conclusion, the results of the present study showed that a coronectomy of an impacted mandibular third molar affected the OHRQoL of patients, particularly in the first three postoperative days. This information should be considered, when assisting patients in planning their schedules and preparing themselves psychologically. A coronectomy seems to have a greater impact on the OHRQoL than the total surgical removal of mandibular third molars.

## References

[1] Queral-Godoy E, Valmaseda-Castellón E, Berini-Aytés L, Gay-Escoda C (2005). Incidence and evolution of inferior alveolar nerve lesions following lower third molar extraction. Oral Surg Oral Med Oral Pathol Oral Radiol Endod.

[2] Valmaseda-Castellón E, Berini-Aytés L, Gay-Escoda C (2001). Inferior alveolar nerve damage after lower third molar surgical extraction: a prospective study of 1117 surgical extractions. Oral Surg Oral Med Oral Pathol Oral Radiol Endod.

[3] Kang F, Xue Z, Zhou X, Zhang X, Hou G, Feng Y (2019). Coronectomy: A useful approach in minimizing nerve injury compared with traditional extraction of deeply impacted mandibular third molars. J Oral Maxillofac Surg.

[4] Leung YY (2019). Management and prevention of third molar surgery-related trigeminal nerve injury: time to rethink. J Korean Assoc Oral Maxillofac Surg.

[5] Pogrel MA, Lee JS, Muff D F (2004). Coronectomy: a technique to protect the inferior alveolar nerve. J Oral Maxillofac Surg.

[6] Leung YY, Cheung LK (2009). Safety of coronectomy versus excision of wisdom teeth: a randomized controlled trial. Oral Surg Oral Med Oral Pathol Oral Radiol Endod.

[7] Hatano Y, Kurita K, Kuroiwa Y, Yuasa H, Ariji E (2009). Clinical evaluations of coronectomy (intentional partial odontectomy) for mandibular third molars using dental computed tomography: a case-control study. J Oral Maxillofac Surg.

[8] Cilasun U, Yildirim T, Guzeldemir E, Pektas ZO (2011). Coronectomy in patients with high risk of inferior alveolar nerve injury diagnosed by computed tomography. J Oral Maxillofac Surg.

[9] Monaco G, Vignudelli E, Diazzi M, Marchetti C, Corinaldesi G (2015). Coronectomy of mandibular third molars: A clinical protocol to avoid inferior alveolar nerve injury. J Craniomaxillofac Surg.

[10] Kouwenberg AJ, Stroy LP, Rijt ED, Mensink G, Gooris PJ (2016). Coronectomy of the mandibular third molar: Respect for the inferior alveolar nerve. J Craniomaxillofac Surg.

[11] Leung YY, Cheung LK (2016). Long-term morbidities of coronectomy on lower third molar. Oral Surg Oral Med Oral Pathol Oral Radiol.

[12] Long H, Zhou Y, Liao L, Pyakurel U, Wang Y, Lai W (2012). Coronectomy vs. total removal for third molar extraction: a systematic review. J Dent Res.

[13] Frenkel B, Givol N, Shoshani Y (2015). Coronectomy of the mandibular third molar: a retrospective study of 185 procedures and the decision to repeat the coronectomy in cases of failure. J Oral Maxillofac Surg.

[14] Tuk JG, Lindeboom JA, Sana F, van Wijk AJ, Milstein DMJ (2019). Alveolar Iodine Tampon Packing Reduces Postoperative Morbidity After Third Molar Surgery. J Oral Maxillofac Surg.

[15] Wijk van AJ, Kieffer JM, Lindeboom JA (2009). Effect of Third Molar Surgery on Oral Health-Related Quality of Life in the First Postoperative Week Using Dutch Version of Oral Health Impact Profile-14. J Oral Maxillofac Surg.

[16] Kieffer JM, van Wijk AJ, Ho JP, Lindeboom JA (2012). The internal responsiveness of the Oral Health Impact Profile-14 to detect differences in clinical parameters related to surgical third molar removal. Qual Life Res.

[17] Smith HS (2006). Arachidonic acid pathways in nociception. J Support Oncol.

[18] Fillingim RB, King CD, Ribeiro-Dasilva MC, Rahim-Williams B, Riley  JL 3rd (2009). Sex, gender, and pain: a review of recent clinical and experimental findings. J Pain.

[19] Bartley EJ, Fillingim RB (2013). Sex differences in pain: a brief review of clinical and experimental findings. Br J Anesth.

[20] Gordon NC, Gear RW, Heller PH, Paul S, Miaskowski C, Levine JD (1995). Enhancement of morphine analgesia by the GABA agonist baclofen. Neuroscience.

[21] Kaiko RF, Wallenstein SL, Rogers AG, Houde RW (1983). Sources of variation in analgesic response in cancer patients with chronic pain receiving morphine. Pain.

[22] Verrips GH (2011). A better life by means of a healthy mouth?. Ned Tijdschr Tandheelkd.

[23] Manor Y, Bader A, Chaushu G, Haim D, Manor A, Gultekin A (2016). How Patients Percept Their Recovery Following Impacted Mandibular Third Molar Coronectomy. J Craniofac Surg.

[24] Zola M (2010). Re: M. Sencimen, et al: Is endodontic treatment necessary during coronectomy procedure?. J Oral Maxillofac Surg.

[25] Renton T, Hankins M, Sproate C, McGurk M (2005). A randomised controlled clinical trial to compare the incidence of injury to the inferior alveolar nerve as a result of coronectomy and removal of mandibular third molars. Br J Oral Maxillofac Surg.

